# Investing for Impact: The Global Fund Approach to Measurement of AIDS Response

**DOI:** 10.1007/s10461-016-1620-6

**Published:** 2016-11-28

**Authors:** Suman Jain, Nathalie Zorzi

**Affiliations:** Monitoring and Evaluation and Country Analysis Team, The Global Fund to Fight AIDS, Tuberculosis and Malaria, Chemin de Blandonnet, 8, 1214 Vernier-Geneva, Switzerland

**Keywords:** Monitoring, Evaluation, HIV, TB, Malaria

## Abstract

The Global Fund raises and invests nearly US$4 billion a year to support programs run in more than 140 countries. The Global Fund strategy 2012–2016 is focused on “Investing for Impact”. In order to accomplish this, timely and accurate data are needed to inform strategies and prioritize activities to achieve greater coverage with quality services. Monitoring and evaluation is intrinsic to the Global Fund’s system of performance-based funding. The Global Fund invests in strengthening measurement and reporting of results at all stages of the grant cycle. The Global Fund approach to measurement is based on three key principles—(1) simplified reporting: the Global Fund has updated its measurement guidance to focus on impact, coverage and quality with the use of a harmonized set of indicators. (2) Supporting data systems—based on a common framework developed and supported by partners, it promotes investment in five common data systems: routine reporting including HMIS; Surveys—population based and risk group surveys; Analysis, reviews and transparency; Administrative and financial data sources; and, Vital registration systems. (3) Strengthen data use: the Global Fund funding encourages use of data at all levels—national, subnational and site level. Countries do not automatically prioritize M&E but when guidance, tools and investments are available, there is high level utilization of M&E systems in program design, planning, implementation, and results reporting. An in-depth analysis of the available data helps the Global Fund and countries to direct investments towards interventions where impact could be achieved and focus on target population groups and geographic areas that are most affected.

## Introduction

The Global Fund raises and invests nearly US$4 billion a year to support HIV, tuberculosis and malaria programs in more than 140 countries to accomplish the Global Fund 2012–2016 strategy of “Investing for Impact” [1]. Timely and accurate data are needed to prioritize activities. Data is essential to measure impact at all levels- local, national and global, which in turn generates new investments. Monitoring and evaluation (M&E) is intrinsic to the Global Fund’s system of performance-based funding, which ensures that funding decisions are based on a transparent assessment of results against time-bound targets. To this end, the Global Fund invests in strengthening measurement and reporting of results at all stages of the grant cycle.

Data are shared publicly and with the donors to document progress toward impact and identify areas to improve the Global Fund’s investment strategy.

The Global Fund M&E model was developed to strengthen, and use existing systems; minimize additional reporting burden on recipients; and empower country-level partners [2]. The model also provides transparency on results achieved. Most notably, it is designed in a way that the information from M&E systems would work hand-in-hand with information from financial systems with the aim of implementing a system of results-based disbursements.

Since the beginning, the Global Fund approach to M&E is based on the following three key principles [3]—simplified reporting, supporting data systems and Strengthen data use. The implementation of these principles has evolved over time with the evolving M&E capacities in the countries and the guidance has evolved too.

It reflects the shift in its funding model from funding projects to supporting national programs and increase the focus on improving coverage and assessing impact. This has resulted in a shift away from grant-specific reporting to coordinated data management with partners- using a consistent set of national indicators, collective and sustainable investments in data systems, disaggregation and data use to support clear, strategic programming to achieve coverage and impact.

## Evolution of Monitoring at the Global Fund

From its inception in January 2002, the Global Fund pursued a rounds based funding model whereby the countries submitted proposals every year outlining their need for additional funding to fill gaps in national strategies. The Global Fund adopted a new funding model in 2012, with several new features including, (a) fixed allocation per country resulting in predictable financing, (b) flexible timeline so that countries could apply anytime during the allocation period and (c) enhanced engagement with countries and focus on multi-stakeholder involvement.

The Global Fund’s M&E Strategy was first adopted in October 2003 [4]. It was designed to respond to the strategic information needs of the countries, other partners including donors and stakeholders and the Global Fund. It recommended that the grant funds be used to strengthen national M&E capacities and encouraged joint partner efforts to this effect. The Technical Evaluation Reference Group (TERG) was established in 2004 to support the Global Fund Secretariat’s M&E function [5]. 2004 was a landmark year when most of the systems and processes for performance based funding were put in place following a hasty start up in 2002. During this year, the secretariat operationalized an approach for results reporting and regular *performance based reviews* including strengthening of information systems to make this reporting feasible. It recommended the grant proposals include budget for M&E and set budgetary parameters by endorsing 5–10% of budgets for M&E.

To ensure a transparent and accountable way of continued-funding, a performance-rating system for each grant was developed. It rated grants as A, B1, B2 and C and took into account the programmatic performance (results against set targets) and the financial performance.^1^ The rating together with an evaluation of contextual considerations impacted how much funding a country can access.

**Fig. 1:**
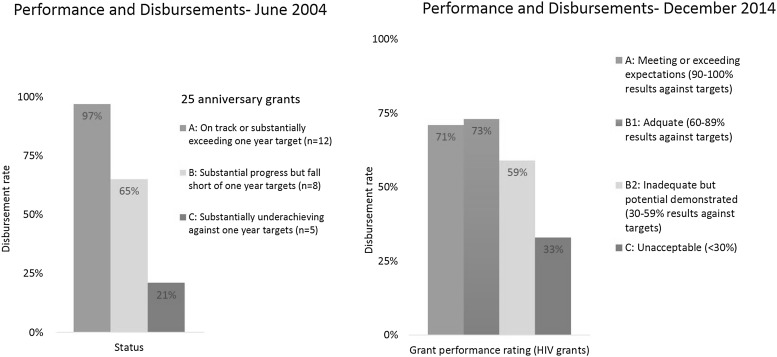
Performance and disbursements in 2004 and 2014. *Source* Global Fund progress report 2004 and grant management platform for 2014

The first Global Fund progress report in 2004, “A Force for Change” [6], analyzed the 25 grants which had been in operation for a year. It showed that the disbursements tend to closely follow performance (Fig. 1). A certain number of grants which were under-performing became the focus of attention by the secretariat to identify the bottlenecks and ways to address them.

By 2005, the *performance based funding* principle was fully embedded in the grant life cycle. It linked funding decisions to the attainment of programmatic results and financial performance.

**Box 1:** Performance based funding framework

The Global Fund system of performance based funding aims to
Ensure money is spent on services for people in need and where impact on the three diseases can be achieved
Relate disbursements to achievement of targets
Provide incentives to focus on results and timely implementation
Identify opportunities early in the Grant lifecycle to expand efforts and address implementation issues
Free up committed resources from non-performing programs for re-allocation to programs where results can be achieved
Develop an evidence base and platform to advocate for sustained and dependable funding

By now the weaknesses in data systems and the uneven capacity of the countries in reporting timely, accurate and complete data was increasingly becoming evident. The data coming from the countries was more focused on inputs and processes and to some extent outputs. The reporting on coverage and the move towards impact and outcome reporting seemed to be a distant reality. Population based surveys were often not planned or delayed due to insufficient resources. Some countries developed parallel reporting systems in order to meet the Global Fund reporting requirements and secure timely disbursements and continued funding for expanding their health programs. It worked well for output reporting but resulted in distortion and inefficiencies for country health information system.

The need for technical assistance and strengthening country capacity for data collection, reporting and analysis was increasingly being felt and several measures were put in place by the Global Fund and its partners.

In 2005, the UNAIDS M&E Technical Assistance System (METAT) was established and supported by a number of partners including the Global Fund, PEPFAR (The United States President’s Emergency Plan for AIDS Relief) and WHO (the World Health Organization). METAT brokered requests for M&E technical assistance from countries and programs with the supply of expertise from technical partners tailored to the local needs.

M&E guidance and materials were developed and updated through the collaborative work of many partners, such as UNAIDS, WHO, UNICEF, PEPFAR, USAID and CDC, other bilateral agencies, non-governmental organizations (NGOs) including MEASURE Evaluation and Family Health International (FHI), and global disease partnerships such as HIV/AIDS 3 by 5 Initiative, Stop TB and Roll Back Malaria. The aim of these guidance materials was to encourage the use of common measures and data systems in order to minimize parallel reporting systems.

The M&E and Data Quality tools were developed and implemented to better identify capacity gaps and guide investments in strengthening national M&E systems and enable assessment of the reliability of the reported data. These tools include the *on-site data verification* (OSDV) implemented every year by the Local Fund Agents^2^ and *data quality audits* (DQA) conducted on a sample of grants by independent bodies.

Despite all these efforts, the report of the five-year evaluation [7] of the Global Fund in 2009 identified inadequate data systems and capacities in the countries. It recommended a further coordinated approach and more systematic investment from partners to strengthen the country health information systems and conduct on-going evaluations.

**Fig. 2:**
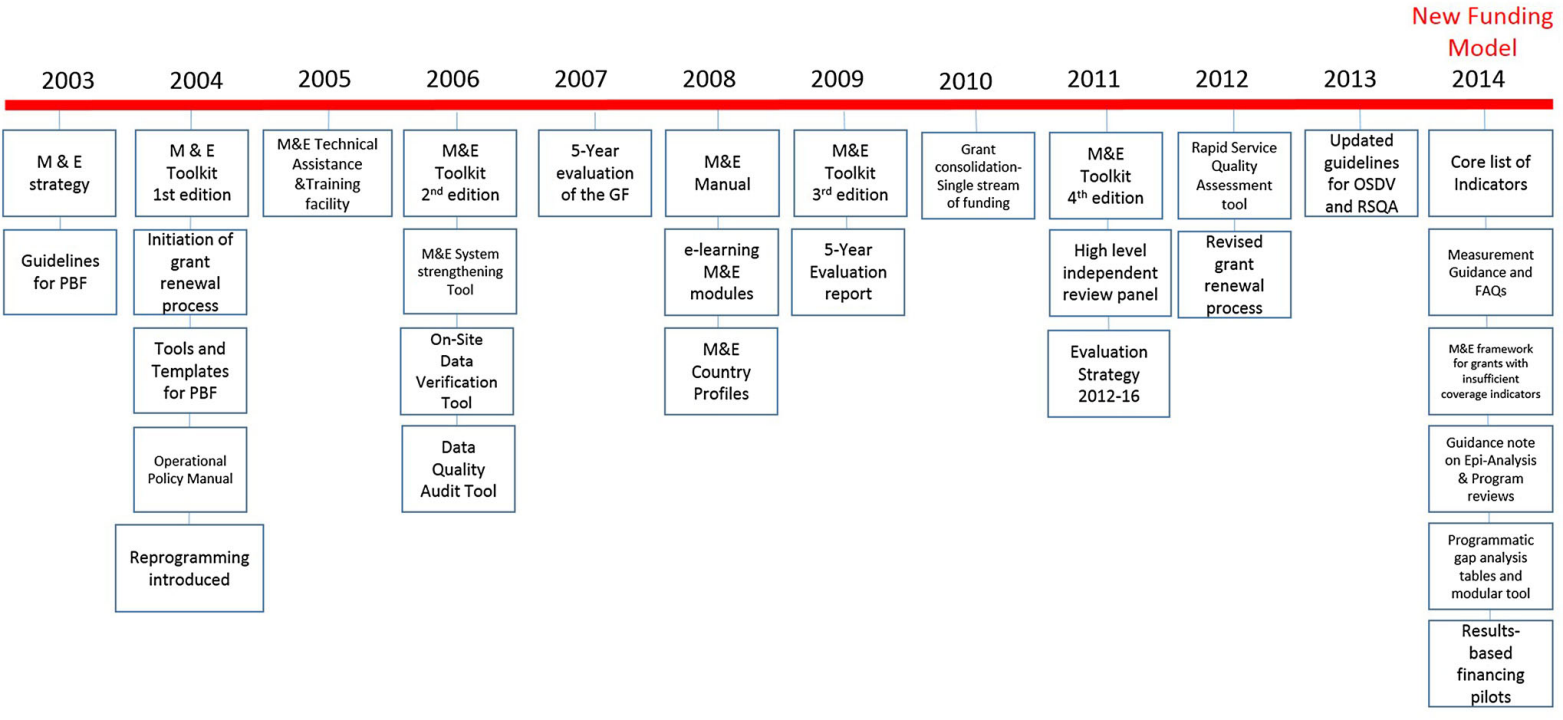
M&E related key milestones (2003–2014) PBF (performance based funding) whereby ongoing financing depends upon performance. The Global Fund uses the principle of performance based funding to ensure that funding decisions are based on a transparent assessment of results against time-bound targets OSDV (on-site data verification) methodology was rolled out by the Global Fund in 2006 to ensure on-site verification of most important programmatic results reported by the grant recipients, and to improve accountability RSQA (rapid service quality assessment), the Global Fund introduced the RSQA tool to have an overall assessmnt of the quality of services delivered under the disease programs. The tool assessed whether health services are implemented according to internationally recognized and evidence-based technical policies and guidelines

In 2011, the high-level independent review panel [8] recommended a “Focus on Outcomes not inputs”. The Panel identified data quality as a risk and recommended investing in country data systems and “paying for baseline data surveys of incidence and prevalence of the three diseases at the country level” and that the Global Fund “mandate and underwrite simple (such as cellphone-based) data tracking and management systems in the field”. M&E related key milestones are shown in Fig. 2 (Table 1).

**Table 1:** Evolution of M&E: 2002–2015

From	To
Customized indicators for each grant	Standardized indicators by epidemic type; Attempts towards harmonization
Input/process indicators in grants	Coverage, outcome and impact indicators
Top 10 indicators	Core list of indicators to track key elements of national response
Multiple standalone grants per country	Single stream of funding supporting the National Strategic Plans
Grant monitoring focusing on the Global Fund supported activities	Tracking progress of national programs towards achieving impact
Data disaggregated by age and sex not collected	Requiring data disaggregation by appropriate categories and analysis of sub-national data for ensuring strategic investments in right populations and geographic areas
Ad hoc M&E investments, not coordinated with other partner efforts	Harmonized efforts with partners towards strengthening 5 key data systems including building analytical capacity
M&E budgets in grants mainly focused on supervision and quarterly meetings	Focused investments towards strengthening data systems, analytical capacity and use of data for planning and management
Focus on government led disease specific interventions and reporting	Inclusion of monitoring and evaluation framework for community, human rights and gender sensitive interventions
M&E and data and service quality assessments seen as Global Fund requirements	Institutionalizing a culture of joint Health Facility Assessments and quality improvement in national programs ensuring country ownership and accountability

## Current Approach to Monitoring: Key Features

The Global Fund’s current funding model was adopted in 2012. It was designed to focus more resources on countries that have the highest disease burden and lowest ability to finance their response to the three diseases.

In order to enable countries to collect, report, analyze and use relevant data for program management and decision making both at local and global level, the Global Fund has streamlined its approach to measurement and M&E to focus on three key principles:

### Simplified Reporting

The Global Fund in collaboration with its partners updated its measurement guidance to focus on impact, coverage and quality with the use of a harmonized set of core indicators [9]. This means reducing process indicators, focusing on a more consistent set of national indicators that are used by all. There are no unique Global Fund indicators. The indicators included in the core list and used for reporting to the Global Fund are taken from the indicators recommended by the partners and are updated regularly to ensure continued alignment with the latest technical guidance.

Another key feature of the revised indicators is improved guidance on the disaggregation of data by age, sex and key population groups. It allows for collecting better information on the populations that are most-at-risk, including their access to key interventions and the outcomes of the interventions for those groups. It aims to provide richer data to support human rights and gender equality and ensure equity of access.

### Supporting Data Systems

The Global Fund supports countries to strengthen their national data systems and capacity to use and evaluate results and impact. It encourages investments in data systems that are agreed and coordinated with partners. Funding from a Board-approved Special Initiatives [10] pool is also available to countries above and beyond their grant funding, in order to bolster specific components of their M&E systems.

Based on a common framework developed and supported by partners, it promotes investment in five common data systems: routine reporting including Health Management Information System (HMIS); Surveys-population based and risk group surveys; Analysis, reviews and transparency; Administrative and financial data sources; and, vital registration systems. In addition, it supports regular data quality reviews and health facility assessments for improving data and program quality.

### Strengthen Data Use

**Fig. 3:**
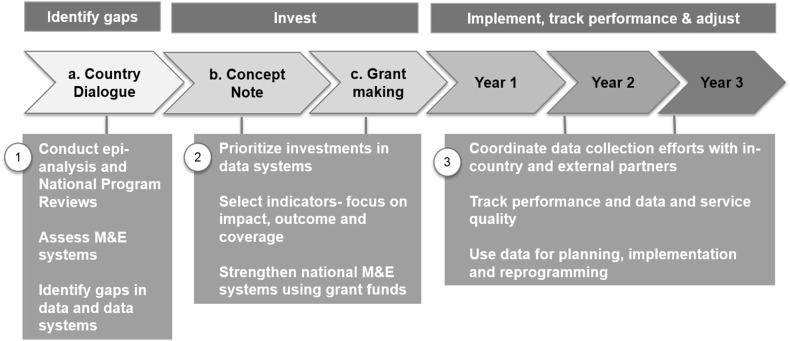
Using data to identify gaps, invest and implement national programs. *Source* The Global Fund

The focus of the Global Fund funding model on robust national strategies and prioritization of investments necessitates that the countries review and analyse the data generated by the programs to ensure that resources are spent where maximum impact can be achieved. It offers countries an opportunity to make strategic investments and to align the Global Fund funding to the country needs. In addition to using data for planning purposes, it promotes the use of data for program management and reprogramming throughout the grant cycle (Fig. 3).

The Global Fund encourages countries to undertake an epidemiological and impact assessment [11] using data disaggregated by age, sex and key populations, both at national and sub-national levels. Such in-depth analysis is then used to develop and/or update the national strategic plans and to inform the funding requests to the Global Fund. This “epi-analysis” stage aims to identify gaps in data and data systems, prioritize key areas for funding, identify key partners and mobilize additional resources.

The Global Fund undertakes regular assessment of coverage and progress towards impact during grant implementation to identify issues and supports reprogramming of its grants so that timely action can be taken to achieve the desired impact. Limited or no progress towards impact and low coverage rates prompt a review of policies, service delivery mechanisms, gaps in funding and other resources followed by development of revised plans for programs to remove bottlenecks.

## Funding for M&E

The Global Fund supports M&E systems strengthening through its disease specific and Health Systems Strengthening grants and through the special initiative^3^ for country data systems (US$17 million approved for 2014–2016 for priority countries^4^). With its continued support, the Global Fund has facilitated the availability and use of data for decision-making, enhancing health systems capacity and resilience.

The cumulative M&E budget in the Global Fund grants from 2003 to 2014 is US$1.5 billion and have increased from US$6.7 million in 2003 to US$212 million in 2014, representing 5 % of the total annual grant budgets [12].

**Fig. 4:**
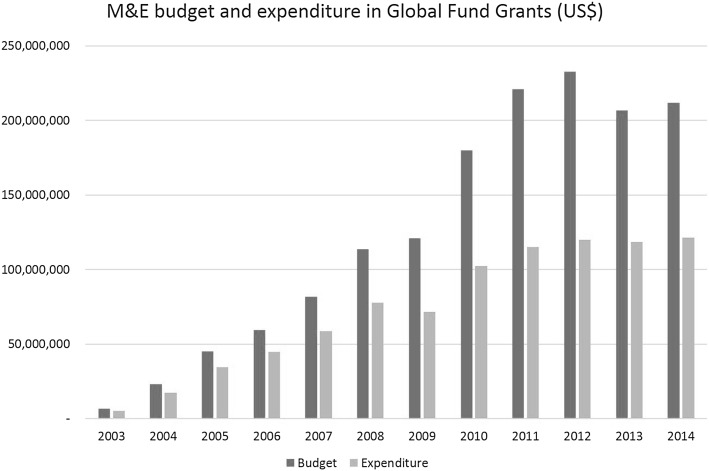
M&E budget and expenditure in Global Fund grants. *Source* The Global Fund grant management platform and enhanced financial reporting system

The expenditure under M&E category in the Global Fund grants during the period 2003–2014 is US$886 million, representing an increase from US$5.2 million to US$121 million and 4% of the total annual expenditures every year [13], Fig. 4. Even though annual M&E budgets in grants increased, these were underspent as often several activities were either delayed or did not take place.

The budget allocated against the M&E modules and interventions currently represents 5.7% of the total budget in the new applications submitted to the Global Fund during 2014 till September 2015 (USD 478 million out of total allocation amount of USD 8.3 billion) [12].

The Global Fund Board, in December 2013 approved additional funding of US$17 million for the Special Initiative on Country Data Systems for the period 2014–2016, to address key data gaps and strengthen underlying data systems needed for assessing impact. All these funds have been approved by December 2016. These amounts are in addition to the budget included in grant applications.

## Key Achievements

The Global Fund’s investments in M&E systems strengthening are synergistic with its core investments in HIV/AIDS, TB and malaria programs. They aim to strengthen national data collection and reporting systems and maximize benefits to the three diseases, as well as improve health systems in general.

Some of the achievements in the country-level M&E systems are attributable to change in the Global Fund approach and operational policies. Since 2012, there has been an increased emphasis on service and data quality, key populations, program reviews, epi-analyses and impact assessments as part of grant management processes. This emphasis has translated to increased attention and an improved focus at the country level, on strengthening M&E systems and positioning programs to achieve impact.

Global Fund investments have strengthened M&E systems in a variety of ways. Some of the achievements over the past two years include the followings:

### Harmonized Set of Indicators

At the beginning of the new funding model in 2014, The Global Fund developed a core set of standard indicators [9] in collaboration with key partners such as WHO, UNAIDS, PEPFAR, World Bank and others. For HIV programs it includes 15 impact and outcome indicators and 25 coverage indicators that cover various epidemic types and key populations. These are taken from the latest recommended partner guidance and are aligned with the global reference list of 100 core indicators for health [14]. These indicators are included in all new grants as per their relevance to the programs. This streamlined and harmonized approach has resulted in more consistent reporting by countries to the different agencies. This has significantly alleviated the reporting burden for countries. This is a key step towards fulfilling the Global Fund commitment to harmonization and alignment and will allow for future joint reviews with partners.

### Key Populations Data Systems

Program planning and delivery of services for key populations are dramatically improved when informed by their population size estimates and geographic location. Also sub-national data on prevalence and behaviors provides increased granularity for better targeting. Ghana and Myanmar, for example, have conducted IBBS across sites in order to improve the understanding of both prevalence and risk factors. In 2013, Sri Lanka successfully completed the mapping and size estimation for sex workers, men who have sex with men, people who inject drugs and beach boys across all 25 districts in the country [15]. The findings provided evidence for improved targeting, M&E systems development, and baseline data for service coverage estimates.

With the Global Fund support, as of July 2016, 49 countries^5^ (including 12 Global Fund priority countries) have nationally adequate estimates for at least two key population groups [17].

### Routine Reporting and HMIS/DHIS

The Global Fund supports the roll-out of comprehensive, nation-wide Health Management Information System, including the District Health Information System (DHIS2)^6^ to strengthen the availability and use of data for planning and decision making at the district and national level. The support in this area includes design of routine information system, health workers training and capacity building, dissemination of M&E standards and guidelines, updating and printing of data collection forms, provision of equipment and technology and access to internet.

DHIS is being used for collecting and analyzing health data for monitoring and reporting in over 50 countries in Africa, Asia and Latin America. Of the 26 priority countries, 17 are using DHIS 2 as a reporting platform with support from the Global Fund. The Global fund has developed a partnership agreement with University of Oslo to ensure continuous technical assistance for implementing DHIS in countries.

**Box 2:** Roll-out of DHIS2 in Zimbabwe [16]: weekly data review has become a management culture

Since 2013, all 10 provinces, all 63 districts, all cities, all of the 6 central hospitals, and all of the 166 admitting hospitals are reporting data using DHIS 2. Over 600 people were trained in DHIS 2 and over 1200 nurses were trained in frontline SMS reporting. The DHIS2 system has resulted in integration of various parallel reporting systems in the country (T5, WDSS, HIV, IMMIS, TB, VHW, EID, HS3/5, Psych, Rehab and IRS). It is linked to SMS reporting and enables weekly and monthly reporting for various purposes
The roll-out of DHIS 2 in Zimbabwe is being supported by the Global Fund HIV grant. The M&E budget in the HIV grant for the period 2014–2016 is US$13 million representing 4.2% of the total grant budget of US$311 million. More than 95% of this is being spent on strengthening the routine reporting system

### Analytical Capacity

The Global Fund is providing financial support and facilitating technical assistance to countries to plan and carry out program reviews including a thorough epidemiological and impact analysis at regular intervals—for example, the mid and end-term reviews of the national strategic plans every 2–3 years.

Between 2012 and 2015, program reviews were completed for 65 country-disease components among the 79 supported by the Global Fund in 26 priority countries. Epidemiological analysis was done for 51 disease components.

### Administrative and Financial Data Source

The Global Fund makes a substantive contribution to the Universal Health Coverage (UHC) agenda by supporting institutionalization of health expenditure tracking systems through its grants and in partnership with the ‘World Health Organization to ensure financial risk protection and effective public spending.

As of date, the Global Fund is supporting National Health Accounts (NHA) institutionalization in forty-six countries. Since 2012 it has co-financed the implementation of NASA in over 20 countries. The Global Fund support is through co-financing of capacity building, on-site technical assistance and operational expenses. It leverages partnerships and pooled resources for a coordinated approach. This has improved unit cost analysis, in-country costing and financing of National Strategic Plans and expenditure reporting including government spending on HIV, expenditures related to key populations and those linked to HIV-specific service delivery. This has helped countries in better understanding of their financial flows, revenue sources and beneficiaries and advocate for leveraging additional government resources.

The Global Fund also supports a number of other initiatives to generate more reliable data of health and disease spending for UHC implementation, disaggregated at sub-national, disease, and beneficiary levels. These include use of data on pharmacy sales to improve estimates of out-of-pocket (OOP) spending by diseases in the Asia–Pacific region and joint expenditure analysis with PEPFAR.

### Mortality Analysis

Currently 15 countries including Tanzania, Nigeria, Ethiopia, Zambia, Zimbabwe, Kenya, Sudan, Bangladesh, Indonesia, India and Viet Nam, are undertaking mapping and analysis of mortality data from various sources. Overall, 17 priority countries are being supported by the Global Fund to carry out mapping of mortality data sources and analysis of mortality and causes of death data from health facilities, community vital registers, sample registration system, surveys and surveillance sources. The Global Fund together with partners has developed a guidance note and a generic protocol for mortality analysis and is facilitating technical cooperation with WHO and other partners.

### Health Facility Assessments

Health facility assessments are a key tool in measuring service availability and readiness, as well as quality of services and data quality. The Global Fund is supporting the priority countries to implement these at either a national or sub-national level. To date, Zambia, Zimbabwe, Philippines, Laos and Benin have approved proposals and 6–8 other countries are in the process of planning an assessment.

An assessment was commissioned following concerns about data and service quality, when Ghana reported 95% retention on antiretroviral therapy (ART) in its Country AIDS Response Progress Report for 2012–2013, despite ART stock-outs. It included a full review of cohort data to determine rates of retention over time rather than aggregate figures at year-end as in the country report. Patient-level retention data was found to be lower than the aggregate data. Service and data quality improvements were identified and included as part of the Global Fund HIV grant application.

## Future Directions

With the new technological advances and updated technical guidance in the field of the three diseases including HIV/AIDS as well as the need for monitoring and reporting on the post 2015 Sustainable Development Goals and the revised Global Targets for ending/eliminating the three diseases, new data requirements have emerged.

In order to meet the current data demands and prepare countries to meet future data requirements, the Global Fund will continue to ensure long-term investments in the routine M&E systems. It will include putting systems in place to track data disaggregated by age, sex and key populations and data at sub-national level. This work has already been initiated in some countries to integrate the surveillance data, case reporting and notifiable disease reporting in HMIS and DHIS. Global Fund will capitalize on the new technological advances and innovation in this area and adapt to new technical guidance and changing priorities.

**Fig. 5:**
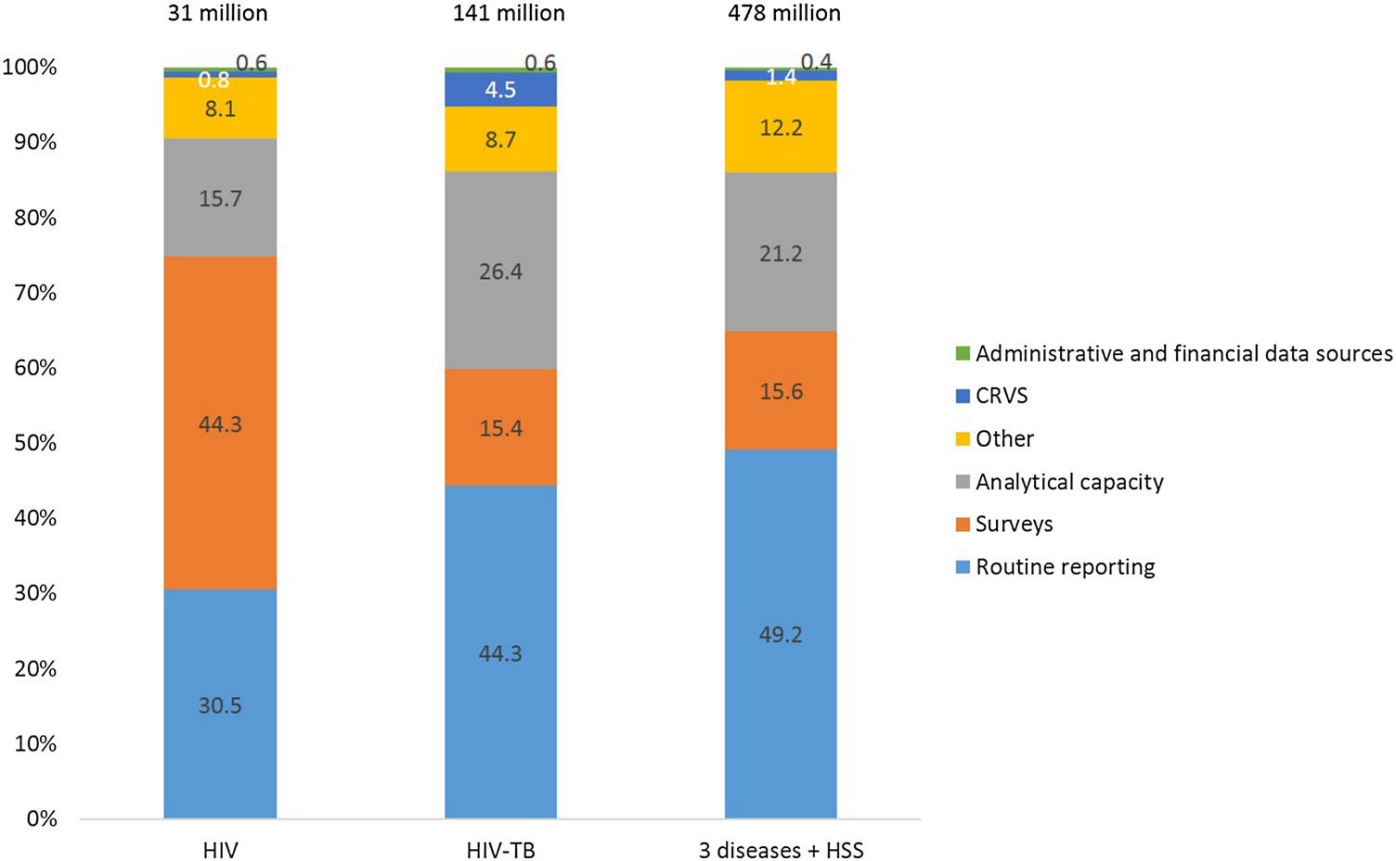
M&E budget request breakdown by category in the concept notes. *Source* The Global Fund grant management platform

The low level of investments in routine reporting systems were identified as a key gap during a mapping exercise conducted by the Secretariat earlier in 2015 in the priority countries. To some extent this is already being remedied in the new Global Fund grants. The concept notes submitted by the countries during 2014–2015 [12] show that approximately 49% of the M&E budgets across all three diseases and HSS were allocated towards strengthening routine reporting systems, followed by 16% for implementing surveys and 21% for data analysis and review. Of the US$141 million budgeted for M&E under the joint TB-HIV concept notes in 2014, 44% were allocated for routine reporting systems, 15% for surveys and 26% for building analytical capacity. Under the standalone HIV applications, out of the M&E budget of US$31 million 30% were for routine systems, 44% for surveys and 16% for building analytical capacity [12], Fig. 5.

In addition to supporting sustainable in-country data systems through collective and coordinated investments in M&E, the key priority going forward will be on supporting in-country utilization of data, from site to district levels, to inform optimal planning, resourcing, reviewing and quality improvement in national health programming. This includes building analytical capacity for better use of data at all levels, in particular for site level management and sub-regional programming. The Global Fund intends to move towards institutionalizing regular epi and program reviews in countries and help inform National Strategic Plan development. It will proactively engage with countries for timely planning of program reviews at least once during the implementation period.

It will continue to support joint health facility assessments for improving data and service quality in order to reduce the burden of frequent and overlapping M&E assessments.

The Global Fund is pursuing strong support for the Call to Action for post-2015 health measurement and accountability and is part of the Health Data Collaborative. It supports a single country platform for information and accountability including alignment of reporting requirements using core indicators.

## Conclusion

The Global Fund investments in M&E have significantly contributed to increasing data availability and quality, data use and ownership at national and local levels. The investments have helped scale up capacities and establish sustainable systems at country level. There is potential for further investments as well as for full utilization of allocated resources for M&E.

Lack of ownership of the M&E assessments and data and service quality as well as of the findings of such assessments by the countries doesn’t allow for sustainable investments in these systems. A common approach to data verification, service quality reviews and health facility assessments at country level is required to build consensus around gaps, required investments and funding sources. In addition, coordinated efforts are needed towards strengthening M&E systems allowing countries to collect and use data to designing, plan, implement, analyze, evaluate and manage their health programs.

The mission and the guiding principles of the Global Fund strategy 2012–2016 makes it clear that it does not operate in isolation in countries and leverages the collective power of all partners. The Global Fund has adopted a contribution model rather than attributing the results to its investments. Ultimately the aim of the Global Fund and its investments in M&E is not merely to inform funding decisions and accountability to donors, but there is an inherent hope and commitment that the data is utilized at national and local levels to ensure that quality services are provided to those in need and no one is left behind.

## Footnotes

1 The grant rating methodology gives a rating of: A = Grant is meeting or exceeding expectations (90 to ≥100% achievement of targets), B1 = Performance is adequate (60–89% achievement), B2 = Performance is inadequate but potential demonstrated (30–59% achievement), and C = Unacceptable (<30% performance).

2 The Global Fund does not have a country-level presence outside of its offices in Geneva, Switzerland. Instead, it hires Local Fund Agents to oversee, verify and report on grant performance.

3 Special Initiatives is a pool of resources approved by the Global Fund Board on 07 March 2014 to support a Humanitarian Emergency Fund; Country Data Systems; Technical Assistance on Community, Rights and Gender; and Enhancing Value for Money and Financial Sustainability of Global Fund Supported Programs. More information on Special Initiatives is available here: http://www.theglobalfund.org/Knowledge/Decisions/GF/B31/DP06/.

4 The Global Fund priority countries are categorized as follows: High Impact Africa 1—Congo (Democratic Republic), Côte d’Ivoire, Ghana, Nigeria, South Africa, Sudan. High Impact Africa 2—Ethiopia, Kenya, Mozambique, Tanzania, Uganda, Zambia, Zimbabwe, Zanzibar, Malawi. High Impact Asia—Bangladesh, Cambodia, India, Indonesia, Myanmar, Pakistan, Philippines, Thailand and Vietnam. Others—Rwanda, Ukraine, Haiti.

5 Bangladesh, Cambodia, Ghana, India, Indonesia, Kenya, Nigeria, Malawi, Philippines, South Africa, Sudan, Tanzania, Thailand, Viet Nam, Afghanistan, Benin, Burundi, Cameroon, Haiti, Lesotho, Madagascar, Mali, Nepal, Sierra Leone, Togo, Ukraine, Azerbaijan, Belarus, Bosnia and Herzegovina, Cape Verde, Dominican Republic, Ecuador, Egypt, Iran, Georgia, Moldova, Guyana, Honduras, Kazakhstan, Kosovo, Kyrgyzstan, Mauritius, Mongolia, Morocco, Panama, Paraguay, Sri Lanka, Timor Leste.

6
https://www.dhis2.org/.
